# Exploration of Mutated Genes and Prediction of Potential Biomarkers for Childhood-Onset Schizophrenia Using an Integrated Bioinformatic Analysis

**DOI:** 10.3389/fnagi.2022.829217

**Published:** 2022-06-16

**Authors:** Fan He, Yu-ming Zhou, Yan-jie Qi, Huan-huan Huang, Lin Guan, Jie Luo, Yu-hang Cheng, Yi Zheng

**Affiliations:** The National Clinical Research Center for Mental Disorders and Beijing Key Laboratory of Mental Disorders, Beijing Anding Hospital, Beijing Institute for Brain Disorders, Capital Medical University, Beijing, China

**Keywords:** *de novo* mutations, exome sequencing, WGSNA, DNA methylation, gene biomarker, childhood-onset schizophrenia

## Abstract

Childhood-onset schizophrenia (COS) is an unusual severe neurodevelopmental disorder of unknown etiology. In this study, we aimed to survey the missense variants in new cases of COS and also identify possible pathology biomarkers for COS. We found one list of mutated genes such as TTN, MUC12, and MUC2, which are the candidates to be involved in the etiology of COS. Next, we used WGSNA to predict COS disease-related genes and identified differential DNA methylation among COS disease groups, COS dangerous groups, and normal groups and found eight methylation sites that can be used as the diagnostic biomarkers. A total of six key genes are obtained through the intersection analysis between weighted correlation network analysis (WGCNA) mode, methylation-related genes, and differentially expressed genes (DGenes). These genes may play important roles in the progression of COS and serve as the potential biomarkers for future diagnosis. Our results might help to design the molecule or gene-targeted drugs for COS.

## Introduction

Schizophrenia (SCZ) is a common and severe mental disorder, with a prevalence rate of about 1% in the general population (Weinberger, 1987; Kirilina et al., 2005). Approximately 1/7–1/3 of this disease begins in children and adolescents. It is estimated that about 13 million people in China suffer from the disease (Ran et al., 2003). Most adult schizophrenia can be traced back to childhood, in which the personality and developmental behavioral traits are different from those of the general population. In addition, the behavioral genetic theory shows that acquired behavior is closely related to genes, so schizophrenia may stem from childhood or earlier abnormalities even if it appears in adulthood (Addington and Rapoport, 2009). So, it makes sense to steer clear of the traditional model of schizophrenia studies focused on adults and instead focus on the specific group of people with childhood-onset schizophrenia (COS) (Wan et al., 2019; Zhang et al., 2020). Since schizophrenia has been described as a kind of disease more than a century ago, revealing that the etiology and pathology of the disease and seeking for effective diagnosis and treatment have been the hot issues concerned by psychiatric departments. Studies in China and abroad suggest that both genetic factors and environmental factors play an important role in the pathogenesis of schizophrenia, which may be caused by the interaction of gene mutation and epigenetic change. The research focusing on copy number variations (CNVs) and family-based relationship has revealed a few genetic variations of COS (Addington et al., 2005; Gornick et al., 2005).

The study of structural variations, such as the microdeletions and microduplications, has shown a higher CNV frequency in patients with COS compared with population controls and adult-onset patients (Awadalla et al., 2010; Ahn et al., 2014). Previous research in patients with schizophrenia found that the rate of *de novo* variants and the potentially harmful *de novo* single base pair variants is higher than expected (McKenna et al., 2010; Girard et al., 2011). Epigenetic modifications are thought to play a role in schizophrenia and other psychiatric diseases with development origins. Recent discovery shows that certain schizophrenia risk loci can affect random variation in gene expression through epigenetic processes. With the advanced development of sequencing techniques, a lot of valuable bioinformatic sources on COS are available from many public databases such as Gene Expression Omnibus (GEO) database and so on.

In this study, we analyzed the *de novo* rare variants in new COS cases by whole-exome sequencing (WES) and try to find out the probable COS candidate genes. To achieve this, we measured the rate of *de novo* variants (DNVs) and used weighted correlation network analysis (WGCNA) to construct a network of co-expressed genes, to describe the correlations among genes across multiple samples. We also checked differential DNA methylation between COS disease groups, COS dangerous groups, and normal groups.

## Materials and Methods

### Sample Overview

A total of 16 cases of COS and 22 cases of normal control children were defined by outpatient clinics or general psychiatric wards and recruited for exome sequencing. The mean age of onset was about 14 years (range, 4–18 years). None of these samples have been exome-sequenced previously. For methylation difference analysis, we used six cases in the disease group, six cases in the dangerous group, and six cases in the normal group (Table 1). The patients have given informed consent, and their anonymity was well preserved. The protocol for the research project has been approved by the Ethics Committee of Beijing Anding Hospital of Capital Medical University (no. 2016103FS-2), and it is conformed to the provisions of the Declaration of Helsinki. All patients and their guardians provided their written informed consent to participate in this study.

**TABLE 1:** Included clinical data and test grouping.

Sample	Gender	Groups	Method
ZHC4	Male	Control^a^	WES^d^
ZHC7	Female	Control	WES
ZHC3	Female	Control	WES
FSZ004	Male	Control	WES
MSZ006	Male	Control	WES
ZHC2	Male	Control	WES
MSZ007	Male	Control	WES
MSZ014	Male	Control	WES
MSZ011	Male	Control	WES
FSZ006	Male	Control	WES
MSZ002	Male	Control	WES
FSZ011	Male	Control	WES
FSZ014	Male	Control	WES
MSZ004	Male	Control	WES
ZHC013	Male	Control	WES
FSZ015	Male	Control	WES
MSZ013	Male	Control	WES
ZHC8	Male	Control	WES
FSZ002	Male	Control	WES
ZHC1	Male	Control	WES
FSZ013	Male	Control	WES
FSZ007	Male	Control	WES
G02005	Female	Case^b^	WES
G01013	Female	Case	WES
G02003	Male	Case	WES
G32	Female	Case	WES
CSZ015	Male	Case	WES
CSZ002	Male	Case	WES
G35	Female	Case	WES
CSZ014	Male	Case	WES
G01008	Male	Case	WES
CSZ006	Male	Case	WES
CSZ007	Male	Case	WES
G43	Female	Case	WES
G18	Male	Case	WES
CSZ004	Female	Case	WES
CSZ011	Female	Case	WES
CSZ013	Male	Case	WES
G025	Female	Dangerous^c^	DMCD^e^
G027	Male	Dangerous	DMCD
G02F049	Male	Dangerous	DMCD
G02M042	Female	Dangerous	DMCD
G35	Male	Dangerous	DMCD
G43	Male	Dangerous	DMCD
CSZ011	Female	Case	DMCD
CSZ013	Male	Case	DMCD
CSZ072	Male	Case	DMCD
CSZ60	Female	Case	DMCD
CSZ79	Male	Case	DMCD
CSZ84	Male	Case	DMCD
ZHC002	Male	Control	DMCD
ZHC007	Female	Control	DMCD
ZHC008	Female	Control	DMCD
ZHC015	Male	Control	DMCD
ZHC018	Female	Control	DMCD
ZHC019	Female	Control	DMCD

*^a^Control, The children in the healthy control group were without mental disorders, and the diagnosis of severe somatic diseases and non-schizophrenia was excluded, which matched the age, sex, education level, and living environment of the study group.*

*^b^Case, The children in the core family of schizophrenia met the diagnostic criteria of schizophrenia in DSM-IV, no other central nervous system diseases and severe somatic diseases, normal intelligence, no history of brain trauma, and drug abuse.*

*^c^Dangerous, The children in the high-risk group of schizophrenia belong to the family of children with schizophrenia, but the patients who are not diagnosed with schizophrenia have no other central nervous system diseases and severe somatic diseases.*

*^d^WES, whole-exome sequencing.*

*^e^DMCD, DNA methylation chip detection.*

### Whole-Exome Sequencing

Exome sequencing was carried out on 38 individuals in the COS cases and performed using SureSelect^XT^ Human All Exon kit V6 (Agilent Technologies, CA, United States). Exome sequence was generated using Illumina HiSeq 2000 in the Genesky Technology company, China. Raw sequencing reads were processed according to the genome analysis toolkit (GATK^1^) best practice guidelines (DePristo et al., 2011; Kakati et al., 2019). Reads were aligned to the human Reference GRCh37.p5 Primary Assembly using the Burrow-Wheeler Aligner (BWA^2^) v0.7.15. Variants were called using GATK haplotype caller (v3.5) and filtered using the GATK variant quality score recalibration tool, VQSR. For all samples that passed quality control, sequence data were generated for a median of 83% of the exome target at ≥10 × coverage.

### Acquisition of Somatic Mutation Data by Maftools

Maftools is an R package published on bioconductor and used specifically for the visualization of information in Mutation Annotation Format (MAF) files. We prepared the MAF of somatic variants and carried out the Maftools to perform the visualization process.

### Gene Expression Omnibus Data Source

We used the keyword ‘‘COS’’ during searching the Gene Expression Omnibus (GEO) database.^3^ The datasets were generated under the following criteria: (1) the datasets contain samples from both normal and patients with COS with necessary clinical features; (2) datasets demonstrate the original expression profiles from microarray that had been corrected and standardized; (3) datasets have enough samples for further analysis. The dataset was selected with GSE19112 using as datasets.

### Weighted Correlation Network Analysis

We used the bioinformatic tool WGCNA to construct the expression patterns of genes from multiple samples and generate the clusters of genes having similar expression patterns by Bioconductor.^4^ It helps the researcher to analyze the correlations between modules and specific phenotypes or features (Langfelder and Horvath, 2008). The soft threshold method was used for expression profiles’ Pearson correlation analysis. In addition, the connection strengths were determined between transcripts to construct the weighted network. We conducted average linkage hierarchical clustering to group transcripts by topological overlap dissimilarity in network connection strengths. To get the correct module number and clarify the gene interaction, we set the limited minimum gene number to 30 of each module and used the 0.25 threshold to merge similar modules.

### Functional and Pathway Enrichment Analysis

Database for annotation visualization and integration discovery (David) (V6.8)^5^ was used to better understand their biological functions. Interested genes were uploaded for Gene Ontology (GO) and Kyoto Encyclopedia of Genes and Genomes (KEGG) pathway enrichment analyses, with cutoff values of *p* < 0.01 and *p* < 0.05 established for significant biological processes and pathways, respectively.

### Protein-Protein Interaction Network and Co-expression Analysis

Interested genes were uploaded to the search tool for the retrieval of interacting genes (STRING) (v11.5)^6^ database. Confidence was set to more than 0.4, and other parameters were set to default. We visualized the gene co-expression network with Cytoscape (v3.73).

## Results

### Landscape of Mutation Profiles in Childhood-Onset Schizophrenia

**FIGURE 1:**
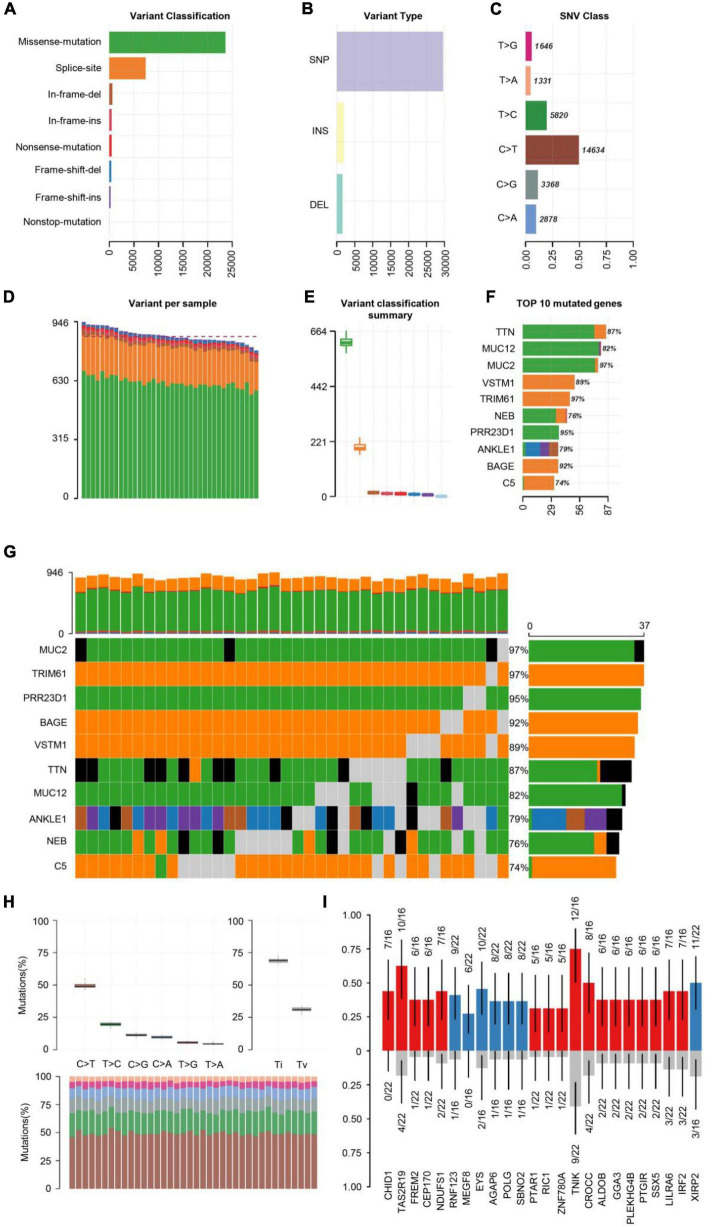
Summary of visualized results of the mutation information by statistical calculations. **(A)** Histogram of the different mutation types’ frequency distribution. The mutation types are classified according to different categories, among which missense mutations account for the largest proportion. **(B)** The frequency distribution of different variant types is divided into three categories: SNP, INS, and DEl. SNP is shown with more frequency. **(C)** The frequency distribution of SNV mutation types is divided into six categories. C > T is the most common in SNV. **(D)** Stacked histograms of different types of variation in each sample. **(E)** Box plot of the distribution of different mutation types per sample. **(F)** Stacked histogram of different types of mutation on the top 10 mutated genes with the highest mutation frequency. **(G)** Oncoplot of the top 10 most frequently mutated genes in COS samples, displaying the distribution of various mutation types in each sample. Each column represents a sample, and each row represents a gene. Mutated genes were shown in colored squares, and no mutated genes were shown in gray squares. **(H)** The transition and crosscut diagrams explain the distribution of SNV in COS with six crosscut and transition events. The mutation spectrum distribution of each sample is shown in the stacked bar graph (bottom). **(I)** The box plots of different allele frequencies between control and cases analyzed by Plotting VAF. Blue represents the number of control samples, red represents the number of disease samples, and gray represents no mutation.

First, the statistics of the diverse mutation types at the sample and gene levels were performed. We carried out the whole-exome sequencing on 38 patients with COS. The results derived from the mutation data with VCF format are visualized by the “maftools” package. Briefly, these mutations are further separated into eight categories based on their impact on protein coding. Among them, the missense mutation accounts for the largest proportion (Figure 1A), single-nucleotide polymorphism (SNP) occurred more frequently than insertion (INS) or deletion (DEl) (Figure 1B). In addition, C > T type was the most common in SNV, which is divided into six categories when considering the combination of mutations in COS (Figure 1C). In addition, we calculated the number of altered bases per sample and displayed the mutation types in diverse colors in the box plot for COS, related to the eight types in Figure 1A and with an average of 866.5 variants in each sample (Figures 1D,E). Finally, we displayed the top 10 mutated genes in COS with ranked percentages, including TTN (87%), MUC12 (82%), MUC2 (97%), VSTM1 (89%), TRIM61 (97%), NEB (76%), PRR23D1 (95%), ANKLE1 (79%), BAGE (92%), and C5 (74%) (Figure 1F). Oncoplot displayed the mutation information of the top 10 most mutated genes in 38 samples, and the different colors with annotations at the bottom stand for the different mutation types (Figure 1G).

Previous research has shown that exploring somatic mutations helps to discover the occurrence and development of schizophrenia (Nishioka et al., 2019). We analyzed the mutation frequency of 10 key genes, with varying degrees of mutation. The plot of transition divides single nuclear variants (SNV) into six categories (Figure 1H). C > T mutation accounted for almost 50% of the total mutations among them. Next, we screened the new key genes which may be related to COS. The box plots showed different allele frequencies analyzed by plotting variant allele frequency (VAF), which helps to quickly estimate the cloning status of top mutant genes (Figure 1I).

### Childhood-Onset Schizophrenia-Related Weighted Correlation Network Analysis Modules and Genes

Then, we analyzed the gene expression in COS cases. In October 2020, we downloaded the GSE19112 gene expression data set obtained from donors with or without COS from GEO and selected the GPL version of the dataset with the largest number of disease samples for further analysis. A total of 76 samples of the dataset GSE19112, 38 patient samples and 38 normal samples, are selected for WGCNA screening of potential genes.

**FIGURE 2:**
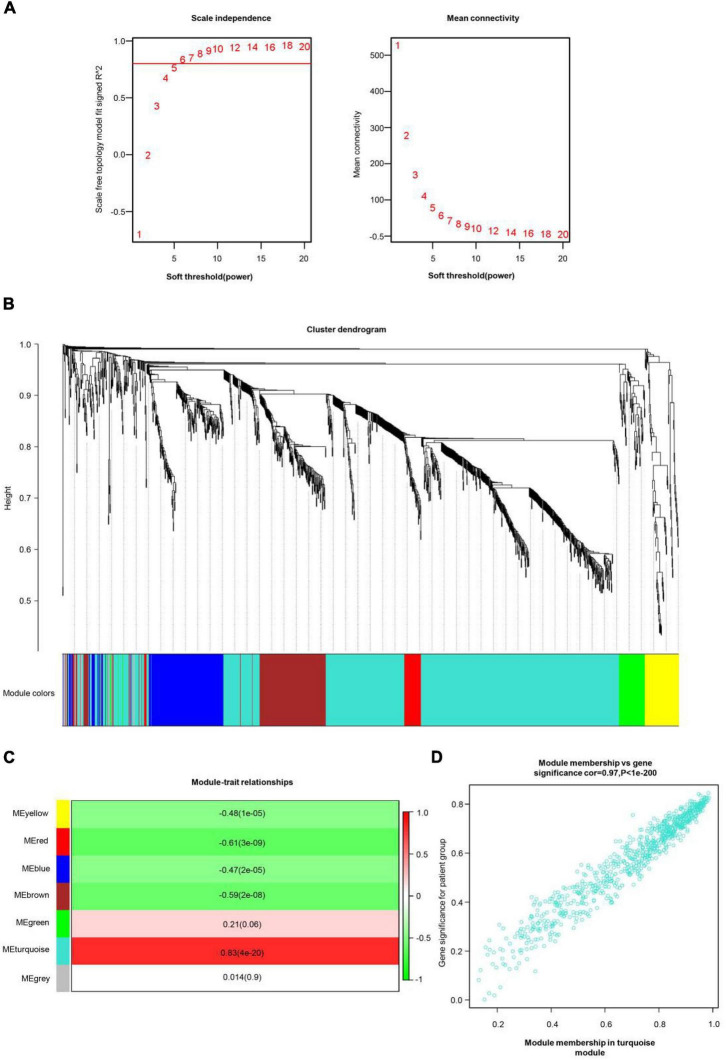
COS-related WGCNA modules and genes. **(A)** The scale independence shown in scale-free topology fit index analysis of soft threshold power (β). The red line represents the correlation coefficient (0.85). Right: Mean connectivity of gene co-expression modules with different soft threshold powers. **(B)** The identification of co-expression module in COS. The branches of the cluster dendrogram symbolized seven gene modules. On the cluster dendrogram, every piece of the leaves corresponds to a diverse gene module. **(C)** The correlation between clinical traits and gene modules was represented by the correlation coefficient in each cell. The most positively and negatively relevant modules were the turquoise and the red, respectively. **(D)** Scatter plot of gene significance (GS) vs. module membership (MM) in the turquoise module.

All genes involved in the GEO dataset have passed WGCNA. The top 25% of genes with the largest variance were preserved for following WGCNA analysis, which ultimately included 1,360 genes and 76 samples. The scale independence was set to be 0.85, and the soft threshold power of matrix transformation was verified to be six for network analysis. The mean connectivity of the co-expression network was high to make sure a scale-free network (Figure 2A). We constructed the co-expression modules and identified seven modules related to COS, which were arbitrarily designated blue (190 genes), brown (176 genes), green (64 genes), gray (32 genes), red (45 genes), turquoise (779 genes), and yellow (74 genes) (Figure 2B). The most common clinical features of COS were selected and linked to gene expression modules founded on the associations between the clinical traits of common expression pattern modules and the eigengenes. The turquoise (*r* = 0.83, *p* = 4e-20) and red modules (*r* = −0.61, *p* = 3e-09) are closely related to the type (Figure 2C).

Then, the scatter plots of gene significance vs. module membership for the turquoise modules were constructed (Figure 2D). It indicated that gene significance for patient group and module membership in turquoise module are positively correlated, cor = 0.97, *p* < 1e-200.

### WGSNA Predicts the Intersection of Childhood-Onset Schizophrenia Disease-Related Genes and Differential Genes

**FIGURE 3:**
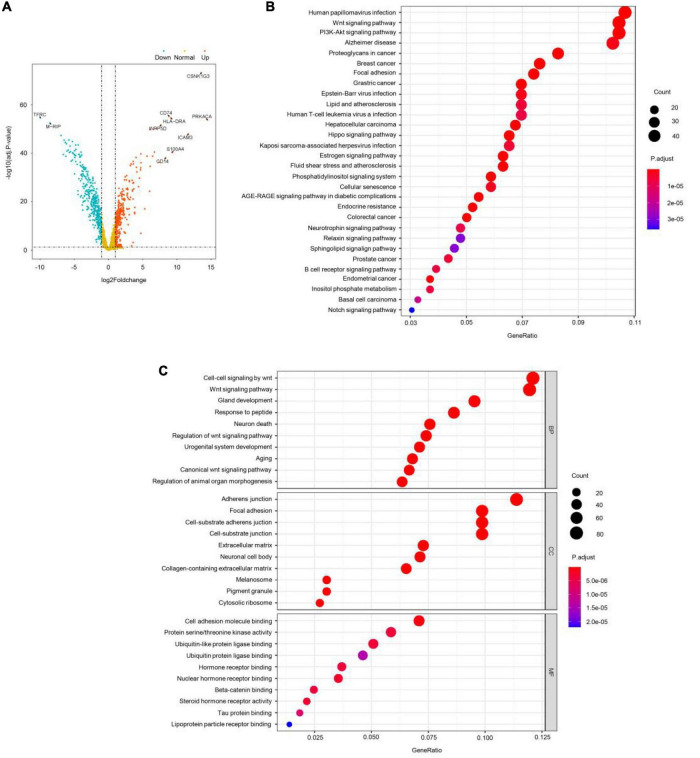
WGSNA predicts the intersection of COS disease-related genes and differential genes. **(A)** X-axis represents log2 fold-changes and Y-axis represents the negative logarithm of the *p*-value to the base 20. Black vertical and horizontal dashed lines reflected the filtering criteria (FC = ±1 and *p*-value = 0.001). **(B,C)** Gene Ontology (GO) enrichment annotations, Kyoto Encyclopedia of Genes and Genomes (KEGG) pathway analysis of the pathological progression of WGSNA predicted the COS disease-related genes and differential genes intersection genes. Significantly enriched pathways featured *p* < 0.001. The analysis was conducted using R clusterProfiler.

Then, GEO GSE19112 data were used for differential expression analysis using ANOVA. A total of 752 significantly differentially expressed genes (DEGs) were identified with cutoff values of *p* < 0.001 and | log2(fold-change) | > 1 (Figure 3A), including 351 upregulated genes and 401 downregulated genes. Next, the ME turquoise gene set was screened by WGSNA above, and the differential genes were intersected and used for GO enrichment analysis. These DEGs were mainly involved in the pathological progress of COS (Figures 3B,C). The most important KEGG pathway terms showed that DEGs were mainly enriched in human papillomavirus infection, wnt signaling pathway, and PI3K-Akt signaling pathway. Considering the BP criterion, DEGs were mainly rich in BPs related to the regulation of cell–cell signaling by wnt and wnt signaling pathway. Considering the CC criterion, the DEGs were mainly enriched in adherens junction. Considering the MF criterion, the DEGs were mainly rich in cell adhesion molecule binding and protein serine–threonine kinase activity.

### Identification of Differential Methylation Sites

**FIGURE 4:**
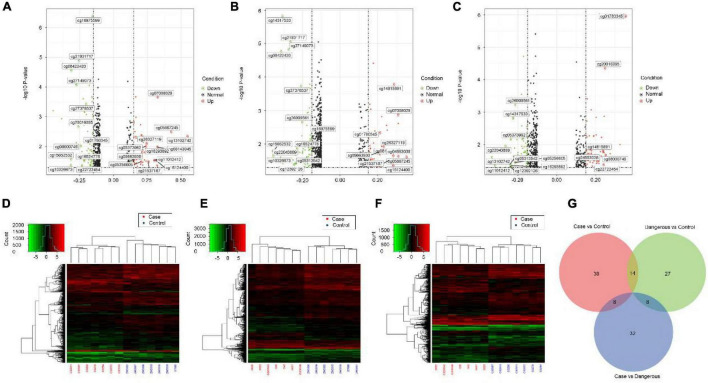
Volcano plots and heatmaps of differential methylation sites. Volcano plots of differential methylation sites were shown in panel **(A)** disease vs. normal, **(B)** dangerous vs. normal, and **(C)** disease vs. dangerous. Red and green points correspond to methylation difference (methylation diff > 0.2 and methylation diff < –0.2) up and down, respectively, and indicate *p*-value < 0.05. The heatmaps of differential methylation sites were shown in panel **(D)** disease vs. normal, **(E)** dangerous vs. normal, and **(F)** disease vs. dangerous. Hierarchically clustered, each column represents a sample, and every row represents a methylation site. The methylation value for each row was normalized by the z-score. Red indicates high relative methylation and green indicates low relative methylation. **(G)** Venn diagram of number of differential methylation sites in three groups. Overlapping sets show the differential methylation sites in two or three comparison pairs.

We also performed genome-wide DNA methylation profiling and the differential DNA methylation analysis in COS cases. Differential analysis of methylation sites in disease samples compared with normal led to the identification of 52 differential methylation sites, named disease vs. normal, including 27 upregulated and 25 downregulated sites. On the other hand, analysis in dangerous samples compared with normal led to the identification of 49 differential methylation sites (named dangerous vs. normal, including 20 upregulated and 29 downregulated sites). Analysis in disease samples compared with dangerous led to the identification of 48 differential methylation sites (named disease vs. dangerous, including 24 upregulated and 24 downregulated sites). We selected significantly upregulated and downregulated methylation sites to plot their differentially degree patterns on volcano plots and heatmaps (Figures 4A–F). Next, we made the intersection analysis between disease vs. normal, dangerous vs. normal, and disease vs. dangerous groups and got eight differential methylation sites, which are used as the key research objects (Figure 4G).

### Function Enrichment Analysis of Differential Methylation Genes

**FIGURE 5:**
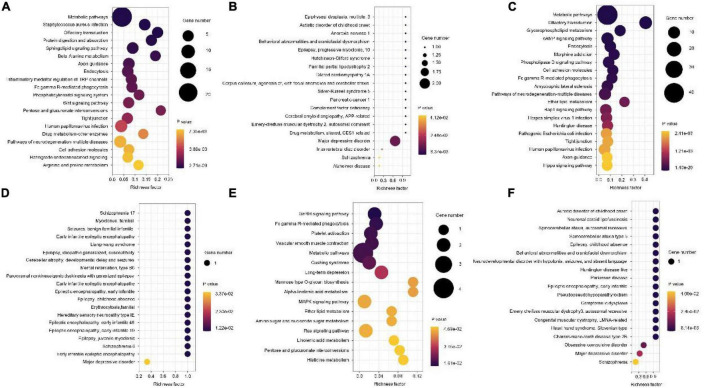
Kyoto Encyclopedia of Genes and Genomes (KEGG) pathway and OMIM analysis of the pathological progression of methylation-related genes in disease vs. normal group **(A,B)**, dangerous vs. normal group **(C,D)**, and disease vs. dangerous group **(E,F)**. Significantly enriched pathways featured *p* < 0.001. The analysis was conducted using R clusterProfiler.

Next, GO enrichment analyses for differential methylation genes were performed. The most significant GO terms in each group are presented in Figure 4. For disease vs. normal group, the differential methylation genes were mainly enriched in KEGG related to the metabolic pathways and enriched in OMIM related to major depressive disorder (Figures 5A,B). For dangerous vs. normal group, the differential methylation genes were mainly enriched in the metabolic pathways and olfactory transduction when considering the KEGG criterion and related to some mental pathways when considering the OMIM criterion (Figures 5C,D). In addition, for disease vs. dangerous group, the differential methylation genes were mainly enriched in KEGG related to the metabolic pathways and enriched in OMIM related to some mental pathways (Figures 5E,F).

### Protein-Protein Interaction Network Construction and Hub Genes Identification

**FIGURE 6:**
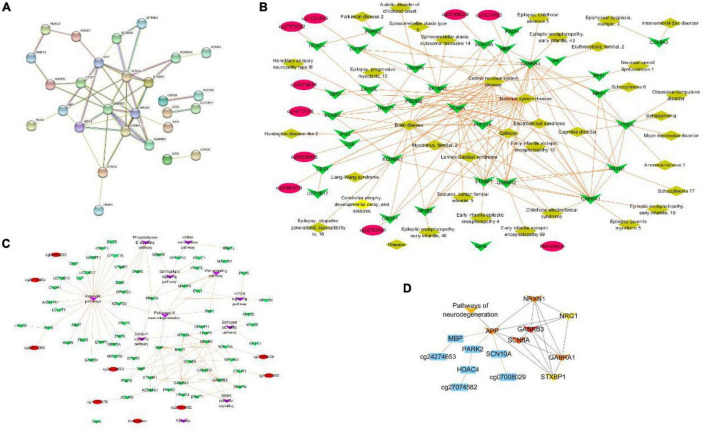
PPI network construction and hub genes identification. **(A)** The protein–protein interaction (PPI) network. **(B)** Methylation-gene-disease network. **(C)** Methylation-gene-pathway network. **(D)** The hob network by CytoHubba.

To further explore the most significant clusters of methylation-related genes, we performed a protein-protein interaction (PPI) network analysis. A confidence score of > 0.7 was set as the threshold and protein nodes that did not interact with other proteins were removed. A total of 28 of 52 methylation-related genes were mapped into the PPI network complex, and the PPI network data file of STRING was imported into Cytoscape. In this network, with a degree of > 5, 15 nodes were chosen as hub nodes, including 12 genes (3 methylation-related genes). The results are presented in Figure 6A. At the same time, we used GeneCodis4 to enrich OMIM and drew methylation-gene-disease network shown as Figure 6B. We also used clusterProfiler to enrich KEGG pathway and draw methylation-gene-pathway network through Cytoscape 3.8.0 as shown in Figure 6C. Finally, we used CytoHubba plugin to screen out hub methylation sites, hub genes, and pathways, as shown in Figure 6D.

### Analysis of Methylation as the Diagnostic Actor

**FIGURE 7:**
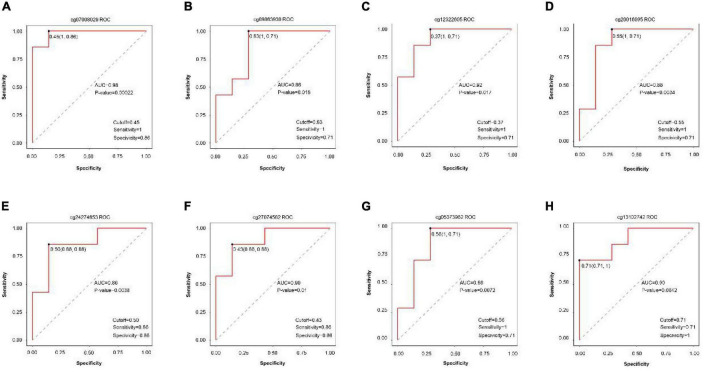
ROC curve comparing sensitivity and specificity of gene methylation panels in disease vs. normal groups. **(A)** cg07008029, **(B)** cg0986930, **(C)** cg12322605, **(D)** cg20016095, **(E)** cg24274653, **(F)** cg27074582, **(G)** cg05373962, and **(H)** cg13102742.

Receiver operating characteristic curves were generated according to the data of disease vs. normal groups methylation and found eight methylation sites that can be used as the diagnostic biomarkers: cg07008029, cg0986930, cg12322605, cg20016095, cg24274653, cg27074582, cg05373962, and cg13102742 (Figure 7).

**FIGURE 8:**
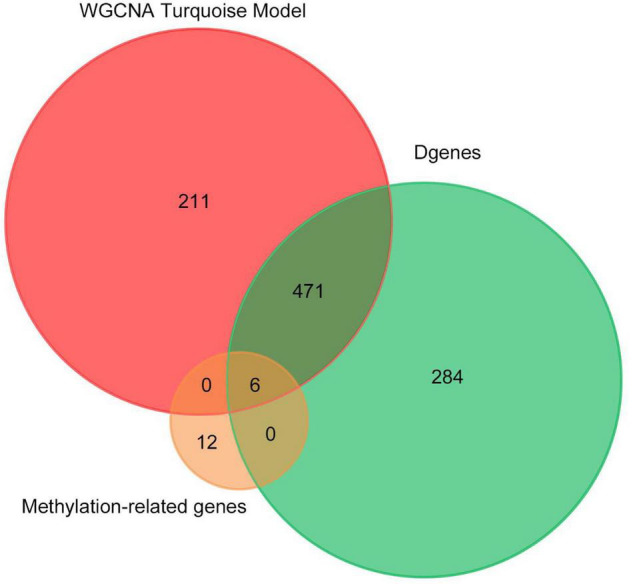
Venn diagram of number of genes in WGCNA turquoise mode, methylation-related genes, and DGenes. Overlapping sets show the number of genes in two or three comparison pairs.

Finally, we made the intersection analysis between WGCNA turquoise mode, methylation-related genes, and differentially expressed genes (DGenes) (Figure 8 and Table 2). A total of six key genes, CYFIP1, LCLAT1, MYH15, SDHAP3, UGT2B17, and HDAC4, are obtained. Importantly, HDAC4 is also found in PPI hub genes.

**TABLE 2:** Genes associated with differential methylation sites.

Probe	BH.adjust	Meth.diff	Chr	Start	End	Gene
cg12322605	0.900526117	−0.220034	15	22904503	22904503	CYFIP1
cg15652532	0.902648545	−0.31672764	2	30669759	30669759	LCLAT1
cg03329597	0.836569569	−0.27489532	3	108125523	108125523	MYH15
cg21931717	0.657885133	−0.25931674	5	1594715	1594715	SDHAP3
cg09139559	0.657885133	−0.2789628	4	69443819	69443819	UGT2B17
cg19481811	0.701258719	−0.44623227	4	69433682	69433682	UGT2B17
cg10632656	0.740084161	−0.38312249	4	69435593	69435593	UGT2B17
cg01617603	0.758659143	−0.3250909	4	69418707	69418707	UGT2B17
cg27074582	0.014882501	−0.23341721	2	240114406	240114406	HDAC4

## Discussion

The exon sequence is only 1% of the total genome but usually associated with disease (Choi et al., 2009; Hedges et al., 2009; Stenson et al., 2009). The presence of the directional genome capture tool makes the exon sequence possible. In recent years, more than 2,000 articles on exome studies have been published in PubMed, and hundreds of diseases are studied deeply by whole-exome sequencing (WES). *De novo* variants (DNVs) which cause disruption of gene function are likely to contribute to the etiology of neuropsychiatric disorders (Girard et al., 2011; O’Roak et al., 2011; Xu et al., 2011; Gilissen et al., 2014). In this study, the association between genes and childhood-onset schizophrenia (COS) was identified by WES from 38 cases. Previous study showed that COS is an early-onset disease, so we hypothesize that DNVs, which are enriched for more harmful and rigorous effects, might play a greater character in COS than in adult schizophrenia. Our study found single-nucleotide polymorphism (SNP) occurred more frequently than insertion or deletion, and C > T was the most common of SNV in COS. We also found one list of top mutated genes such as TTN, MUC12, MUC2, VSTM1, TRIM61, NEB, and PRR23D1, which are the candidates to be involved in the etiology of COS. Among the mutated genes identified, some were previously reported in schizophrenia or other neuropsychiatric disorders through the literature search and seem to be good candidates as COS predisposing genes.

Previous research to identify the compound mutations of SCZ initiated the titin (TTN) gene with the rare protein-altering compound heterozygous mutations in SCZ trios (Li et al., 2020). MUC12, the member of mucins protein family, is important for intestinal integrity and related to Crohn’s disease (CD) and ulcerative colitis (UC) (Buisine et al., 1999; Moehle et al., 2006; Luu et al., 2010). Next, we will further check whether these interesting genes are involved in biological pathways related to COS, especially the early development of the central nervous system, and also validate these results in a larger COS cohort.

As WGCNA is reportedly to be more reliable and more biologically significant than other methods (Chou et al., 2014), we used this method to form the clusters of predictive functionally related genes. In this way, we identified modules and selected genes that might be considered as the biomarkers for the diagnosis and/or treatment of COS. We used WGCNA to construct 7 COS-related co-expression modules containing 1,360 genes identified in 76 samples to determine the relationships between the COS transcriptome and clinic traits. A total of two co-expression modules were strongly associated with the clinical traits of COS. Differential expression analysis with ANOVA identified 752 significant DEGs, including 351 upregulated and 401 downregulated genes. Although WGCNA may not be as effective as other techniques in identifying modules with high functional relevance and biological significance, it is the most widely used for this purpose (Kakati et al., 2019). We used this correlation-based gene screening method to identify candidate biomarkers and/or therapeutic targets for COS.

Methylation of DNA usually occurs in the cytosine 5-carbon position of CpG dinucleotide and appears significant in normal development and diseases. Until now, no studies have checked the differences in DNA methylation in COS. In this research, which is the first to disclose differences in DNA methylation in COS cases, we performed a genome-wide DNA methylation analysis between COS disease groups, COS dangerous groups, and normal groups. The differential methylation genes were mainly enriched in KEGG related to the metabolic pathways, and eight methylation sites were found to be used as the diagnostic biomarkers. Finally, six key genes, such as CYFIP1, LCLAT1, MYH15, SDHAP3, UGT2B17, and HDAC4, are obtained through the intersection analysis between WGCNA turquoise mode, methylation-related genes, and DGenes. Significantly, HDAC4 is also found in PPI hub genes.

The histone deacetylases (HDACs) participate in the pathological and physiological conditions by regulating the histone acetylation status. The histone deacetylase 4 (HDAC4) is a member of the HDAC family lacking HDAC activity. HDAC4 could interact with signal transduction molecules, transcription factors, and HDAC3 to regulate the transcription of genes in neurodevelopment, neuronal survival, and synaptic plasticity. The homeostasis of HDAC4 highly expressed in the brain is essential for maintaining cognitive function. Previous research indicates that the abnormal HDAC4 expression plays a key role in the cognitive impairment of several brain diseases, such as mental disorders and neurodegenerative diseases. Cognitive disorders are common in patients with schizophrenia, especially memory and learning impairment. Some evidence suggests that HDAC4 may be related to the pathology of schizophrenia (Wu et al., 2016). Other data support the idea that inhibition of HDACs by small molecules might offer a therapeutic alternative for the treatment of many symptoms linked with schizophrenia, especially cognitive deficits (Weïwer et al., 2013). In the future, it will be important to study the role of HDAC isoforms in schizophrenia and to emphasize the technology to develop selective inhibitors of these isoforms as the potential treatments for schizophrenia.

In our study, we analyzed the mutation profiles using the WES data and found one list of mutated genes. We also used WGCNA to construct a network of co-expressed genes and identified differential DNA methylation. A total of six key genes are obtained through the intersection analysis between WGCNA mode, methylation-related genes, and DGenes. These genes may play important roles in the progression of COS and serve as the potential biomarkers for future diagnosis. Our results could help to design the molecule or gene targeted drugs for COS. This study also has limitations. Because the children’s sample is very difficult to collect, we did not get a large sample size and needed to perform the repeated studies containing larger samples. Based on the present data, we speculate the disruption of gene function caused by DNVs and/or DNA methylation difference in COS could affect the expression of functional genes, such as HDAC4, related to the etiology of neuropsychiatric disorders, especially schizophrenia. To test this hypothesis, we will perform further research to confirm the links between the disruption of regulatory gene function and the expression of functional genes in COS progress with larger samples.

## Data Availability Statement

The original contributions presented in this study are publicly available. These data can be found here: NCBI, PRJNA83030 and PRJNA828362.

## Ethics Statement

The studies involving human participants were reviewed and approved by the Ethics Committee of Beijing Anding Hospital of Capital Medical University. Written informed consent to participate in this study was provided by the participants’ legal guardian/next of kin.

## Author Contributions

Y-HC and YZ conceived and planned the experiments. Y-JQ performed the experiment and acquired the data. H-HH analyzed and interpreted the data. FH drafted the manuscript. Y-MZ revised the manuscript. All authors provided critical feedback, helped in shaping the research, analysis, and manuscript, and read and approved the final submitted manuscript.

## Conflict of Interest

The authors declare that the research was conducted in the absence of any commercial or financial relationships that could be construed as a potential conflict of interest.

## Publisher’s Note

All claims expressed in this article are solely those of the authors and do not necessarily represent those of their affiliated organizations, or those of the publisher, the editors and the reviewers. Any product that may be evaluated in this article, or claim that may be made by its manufacturer, is not guaranteed or endorsed by the publisher.

## Footnotes

1
https://software.broadinstitute.org/gatk/best-practices/

2
http://bio-bwa.sourceforge.net/

3
http://www.ncbi.nlm.nih.gov/geo/

4
http://bioconductor.org/biocLite.R

5
https://david.ncifcrf.gov/

6
https://string-db.org/
